# Peripheral clock disruption and metabolic disease: moving beyond the anatomy to a functional approach

**DOI:** 10.3389/fendo.2023.1182506

**Published:** 2023-05-22

**Authors:** Gabriella M. Marino, Deanna M. Arble

**Affiliations:** Department of Biological Sciences, Marquette University, Milwaukee, WI, United States

**Keywords:** circadian, metabolism, clocks, bmal1, mouse models, endocrinology

## Abstract

Sleep and circadian disruption are associated with an increased risk of metabolic disease, including obesity and diabetes. Mounting evidence indicates that misaligned and/or non-functional clock proteins in peripheral tissues critically contribute to the presentation of metabolic disease. Many of the foundational studies which led to this conclusion have focused on specific tissues such as the adipose, pancreas, muscle, and liver. While these studies have greatly advanced the field, the use of anatomical markers to manipulate tissue-specific molecular clocks may not be representative of the circadian disruption that occurs within the clinical population. In this manuscript, we argue that investigators can gain a better understanding of the consequences of sleep and circadian disruption by targeting groups of cells with a functional relationship, even if those cells go beyond anatomical boundaries. This approach is especially important when considering metabolic outcomes which rely on endocrine signaling molecules, such as leptin, that have multiple sites of action. Through the review of several studies, as well as our own work, this article reframes peripheral clock disruption from a functional approach. We additionally present new evidence that disruption of the molecular clock within all cells expressing the leptin receptor affects leptin sensitivity in a time-dependent manner. Taken together, this perspective aims to provide new insight into the mechanisms leading to metabolic disease associated with circadian disruption and various sleep disorders.

## 1 Peripheral clocks in health and metabolic disease

Most organisms express 24-hr rhythms in molecular, physiological, and behavioral phenomena. These patterns, also known as *circadian rhythms*, are organized by an endogenous clock located in the suprachiasmatic nucleus (SCN) of the anterior hypothalamus. The SCN utilizes light cues detected by the retina to entrain its endogenous clock to the 24-hr solar day. Signals derived from the SCN, in turn, synchronize a ~24hr, molecular transcriptional-translational feedback loop of core clock genes within individual peripheral tissues. Note here, the word “peripheral” is not restricted to cell populations outside the central nervous system, but instead indicates any cell outside the SCN. Over the past decades, there has been increased recognition that well-aligned clocks are important for metabolic health. Often called *circadian disruption* or *circadian misalignment*, endogenous circadian rhythms can become out-of-phase with respect to the external light:dark cycle and/or out-of-phase with respect to other circadian rhythms within the body. For example, individuals who are awake and active during the night (*e.g*., third shift or night-shift workers) are more likely to have circadian rhythms that are out-of-phase with the solar day and are at increased risk of obesity, diabetes, and cardiovascular disease (1). Individuals who skip breakfast or eat most their daily calories in the evening are more likely to exhibit internal circadian misalignment, where there is a mismatch among an individual’s peripheral clocks. These individuals are at higher risk of obesity and diabetes (2). Observations such as these have propelled laboratory studies which have further demonstrated that individuals on non-24hr days (3) and animals with genetic perturbation to molecular circadian rhythms (4, 5) exhibit increased weight gain and glucose intolerance. An overarching hypothesis is that the disruption of peripheral clocks accounts for the observed impairments in metabolic health, and that the proper alignment of peripheral clocks may have beneficial effects. However, the specific cell population(s) involved, and the mechanism by which they become disrupted, remains an open research question. To understand the context of this question and to better identify future research avenues, it is essential to review two key milestones which have shaped our understanding of peripheral clocks in the context of metabolic disease.

### 1.1 *Per::Luc* models

In the early 2000s, two transgenic models were created which enabled researchers to view the circadian oscillation of gene expression in real-time. Both models utilized the bioluminescent gene, *Luciferase* (*Luc*), expressed under the promotor of a core circadian gene, *Period1* (*Per1*) (6) or *Period2* (*Per2*) (7). Using these *Per*::*Luc* models, SCN and peripheral tissue cultures were found to exhibit rhythmic clock gene expression for multiple weeks *ex vivo*. *Per*::*Luc* models additionally provided strong evidence that peripheral tissues, such as the adipose, pancreas, muscle, and liver, exhibit rhythmic clock gene expression profiles that differed from the SCN and from each other. The complexity of peripheral clocks deepened with multiple observations that peripheral clock rhythms did not re-entrain to shifts in the light:dark cycle at the same speed, and instead had independent rates of synchronization. In response to a 6-hr phase delay, for example, the SCN reorganizes to the new light:dark cycle quickly while other tissues, such as the liver, take more than 5 days to fully re-align their clocks (6). This helped to form the idea of internal disruption – that circadian disruption can not only occur between an animal and the light:dark cycle, but can also occur among peripheral clocks. These *Per::Luc* models, combined with the early studies noting rhythm and re-entrainment differences between peripheral tissues, inadvertently began to shape an “anatomical” view such that peripheral clocks were often associated and distinguished by the tissue they were collected from as opposed to specific cell populations.

### 1.2 Food anticipatory activity and the food entrainable oscillator

In addition to providing foundational evidence regarding peripheral clock oscillations, the *Per::Luc* models also accelerated our understanding of how food as an entraining cue synchronized peripheral clocks independent of the SCN. In 1979, Stephan demonstrated that SCN-lesioned rats could anticipate meals when food was offered every 24-hrs during a 4-5 hour feeding window (8). Anticipation was measured by an increase in locomotor activity 1-2 hours prior to the scheduled food appearance. This phenomenon was named Food Anticipatory Activity (FAA) and led to the hypothesized Food Entrainable Oscillator (FEO) – a theoretical clock that was synchronized by food instead of light. Later, this classic restricted feeding protocol was used in conjunction with the *Per2::Luc* model to demonstrate that, not only was restricted feeding capable of driving FAA, it also synchronized the *Per2* rhythms of peripheral tissues. Notably, clock-controlled genes in the liver, heart, and pancreas largely entrained to food, while the same genes in the SCN remained synchronized to light timing (9). This key finding indicated that internal misalignment could be achieved when food was presented out-of-phase with SCN-dictated feeding periods. Later, investigators observed that mice fed exclusively during the 12-hr light phase (a protocol called *desynchronized* feeding) gained significantly more weight than those fed exclusively during the SCN-dictated dark phase (10). This observation, as well as many others (for review, see 2), support the idea that the internal misalignment of peripheral clocks, as achieved by feeding at the “wrong” time of day, may contribute to metabolic disease. With the knowledge that peripheral tissues exhibit cyclic clock gene expression and that their misalignment from the SCN is associated with metabolic disease, the field turned to tissue-specific manipulations to determine how peripheral clock misalignment might lead to metabolic impairments.

## 2 An anatomical approach to understanding circadian disruption, peripheral clocks, and metabolic disease

In the early 2000s, investigators began manipulating various core clock genes and characterizing the resulting metabolic effects. A mutation in the circadian gene, *Clock*, resulted in a mouse with an approximate ~28-hr endogenous period as well as obesity and decreased insulin production (5). Deletion of the circadian gene, *BMAL1*, disrupted the clock’s molecular transcriptional-translational loop and resulted in a severely affected mouse with a shortened life span, glucose intolerance, and increased adiposity, as well as disrupted heart rate and blood pressure rhythms (4, 11–13). These whole-body genetic manipulations largely mirrored the metabolic outcomes observed in the clinic, including the increased risk of obesity and diabetes observed in night-shift workers as well as in individuals with Night Eating Syndrome (1, 14). Individuals experiencing experimentally-induced circadian misalignment similarly exhibited increased glucose levels as well as reduced leptin and sleep efficiency when meals and sleep time occurred ~12h out-of-phase with their endogenous clock (3, 15, 16). Building on these studies, investigators began utilizing manipulations of the molecular clock within specific tissues to help determine the mechanism(s) by which circadian disruption affects metabolic physiology.

Given that deletion of *BMAL1* can dissolve the molecular clock through the manipulation of a single gene, many investigators focused on this approach to break the molecular clock of target cell populations. A caveat to this approach is that while this leads to molecular arrhythmicity, the lack of *BMAL1* can also impact non-circadian processes that depend on direct interaction with *BMAL1*. Nevertheless, this conditional knockout approach was used with powerful Cre-Flox technology (for review see 17) to produce many influential studies. Notably, the conditional knockout of *BMAL1* selectively in the adipose, pancreas, or skeletal muscle produces metabolic outcomes consistent with those observed in the clinic. Male mice lacking a molecular clock within adipose tissue consume more calories during the light phase, exhibit increased obesity (particularly in response to a high-fat diet), have larger adipocytes, and have elevated leptin and triglyceride levels (18). Mice lacking a molecular clock within the pancreas have normal body weights and feeding patterns, but exhibit predominant changes in glucose homeostasis, including increased non-fasting glucose, decreased glucose tolerance, and decreased glucose-stimulated insulin release (19). Deletion of *BMAL1* from skeletal muscle decreases body fat but impairs glucose regulation as demonstrated by an increase in non-fasting glucose, as well as a decrease in glucose tolerance and muscle-dependent insulin response (20). In contrast, lack of *BMAL1* within the liver has little to no effect on body weight regulation but improves glucose tolerance and decreases non-fasting glucose during the inactive period of male mice (21). Overall, these studies suggest that the adipose peripheral clock is involved in body weight regulation, and likely some feeding behaviors, while the molecular clock of several other peripheral tissues including the pancreas, skeletal muscle, and liver primarily influences glucose regulation. However, it is unlikely that circadian disruption, as it occurs in humans, singularly impacts one tissue. Instead, it is more likely that circadian disruption affects multiple organ systems. This limitation of the tissue-specific approach restricts the ability of these models to unmask the clinical mechanism by which circadian disruption leads to metabolic disease.

## 3 An example of a functional approach: the deletion of *BMAL1* in leptin receptor-expressing cells

While targeting specific tissues has greatly advanced our understanding of how peripheral clocks contribute to metabolic disease, future studies would do well to consider how circadian disruption impacts metabolic disease based on systemic signals and not anatomical boundaries. For example, perhaps circadian disruption affects metabolic health not by being particularly detrimental to one specific tissue but by negatively affecting all cell populations that respond to a specific entraining cue and/or hormone. Indeed, mounting evidence links mistimed meals with adverse metabolic health (for review see 2). This supports the idea that food, as an entrainment cue, has widespread effects on multiple cell populations that collectively contribute to metabolic disease. Moreover, the observation that circulating insulin and leptin rhythms are altered in response to circadian disruption in a manner consistent with metabolic disease supports the notion that an endocrine signal may provide a mechanistic link between circadian rhythms and metabolic health. Therefore, we argue that investigators can gain novel insight into the mechanisms of circadian disruption by targeting cell populations that receive similar entraining signals, even if those cell populations span anatomical borders – a perspective we will refer to as a “functional” approach.

Unfortunately, the field’s current ability to critically evaluate this hypothesis is significantly limited by a lack of understanding of *how* peripheral clocks are synchronized by the SCN and other entraining agents. Indeed, in addition to input by the master clock, it is likely that peripheral clock coordination is a product of both endocrine and exocrine factors, as well as input from the autonomic nervous system (22). Moreover, it is largely unknown *what* peripheral cell populations are principally entrained, and the extent to which they oscillate independently of nearby cells. Indeed, we now know that the tissue-level resolution of the early *Per::Luc* imaging studies does not fully capture the complexity of peripheral clocks. As demonstrated by Petrenko et al., (23) for example, the α and β cells of the pancreas oscillate with different phases. This opens the possibility that any heterogeneous tissue may contain distinct cell populations which differentially respond to synchronizing cues. This also invites the idea that disrupted clock rhythms within a subset of cells, as opposed to the whole tissue, may exert negative effects on whole-body physiology.

**Figure 1:**
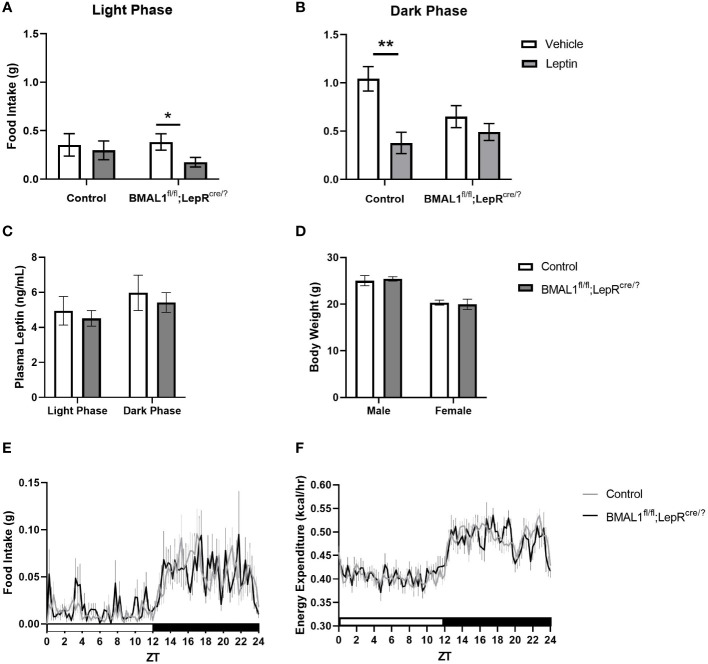
Conditional knockout of *BMAL1* from all leptin receptor-expressing cells increases behavioral leptin sensitivity without affecting circulating leptin, body weight, daily food intake, or energy expenditure. **(A)** In contrast to control, *BMAL1^+/+^*;LepR^cre/?^ mice (n = 11), *BMAL1^fl^* ^/fl^;LepR^cre/?^ mice (n = 12) exhibit a relative increase in leptin sensitivity during the light phase, but a relative decrease in leptin sensitivity during the dark phase. Methods: BMAL1^fl/fl^ mice (purchased from the Jackson Laboratory) and Leptin receptor^cre/cre^ founder mice (provided by Dr. Martin Myers, Jr., University of Michigan) were bred together to create the *BMAL1^fl^* ^/fl^;LepR^cre/?^ and *BMAL1^+/+^*;LepR^cre/?^ mice. Mice were acclimated to indirect calorimetry cages (Sable Systems, North Las Vegas, NV) for at least 24 hours. Following acclimation, mice were fasted for 6 hours and then immediately received an intraperitoneal injection of either mouse leptin (1 mg/kg) or 0.9% saline at either ZT 1 (Light phase) or ZT 11 (Dark phase). Food intake was collected over the subsequent five hours. Injections were counterbalanced over multiple cohorts such that every mouse received leptin and saline injections at each time point. Data was analyzed with SPSS, repeated measures multi-factorial ANOVA. There was a significant main effect of time of day (p < 0.001), and leptin treatment (p = 0.001). There was no main effect of genotype (p = 0.18) or sex (p = 0.49). There were significant interactions between time of day and leptin treatment (p = 0.04), genotype, sex, and leptin treatment (p = 0.04), and time of day, genotype, and leptin treatment (p = 0.02). Pairwise comparisons, as depicted on graph, were made with a Bonferroni *post hoc* test. * p < 0.05; ** p < 0.01. **(C)** Regardless of genotype, plasma leptin exhibited a diurnal variation, with lower levels occurring during the light phase. Lack of *BMAL1* in leptin receptor-expressing cells had no effect on overall leptin levels. Methods: Blood samples were collected from the tail of freely-feeding male and female *BMAL1^fl^* ^/fl^;LepR^cre/?^ (n = 23) and *BMAL1^+/+^*;LepR^cre/?^ mice (n = 28) at 14 to 16 wks of age. Samples were collected at ZT 10 and ZT 22. Plasma leptin was assessed by an ELISA kit (Crystal Chem, Elk Grove, IL) according to the manufacturer’s instructions. Data was analyzed with GraphPad Prism, repeated measures two-way ANOVA. There was a significant main effect of time (p < 0.0001). There was no main effect of genotype (p = 0.62) or interaction effect of time and genotype (p = 0.94). Multiple comparisons were made using Šidák’s *post hoc* test. **(D)** *BMAL1^fl^* ^/fl^;LepR^cre/?^ mice weighed similarly to age- and sex-matched controls (*BMAL1^+/+^*;LepR^cre/?^ mice). Methods: Male and female *BMAL1^fl^* ^/fl^;LepR^cre/?^ (n = 23) and *BMAL1^+/+^*;LepR^cre/?^ (n = 28) mice were weighed at 13 to 23-wks of age. Data was analyzed with GraphPad Prism, repeated measures two-way ANOVA. There was a significant main effect of sex (p < 0.0001). There was no main effect of genotype (p = 0.98) or interaction effect of time and genotype (p = 0.63). Multiple comparisons were made using Šidák’s multiple comparison *post hoc* test. **(E, F)**. *BMAL1^fl^* ^/fl^;LepR^cre/?^ mice consumed similar quantities of food and demonstrated similar caloric expenditure over the light:dark cycle compared to age- and sex-matched controls (*BMAL1^+/+^*;LepR^cre/?^ mice). Methods: After acclimation to indirect calorimetry cages (Sable Systems, North Las Vegas, NV), food intake and energy expenditure were collected over the subsequent 24 hours. Data was analyzed with GraphPad Prism, repeated measures two-way ANOVA. There was a significant main effect of time (p < 0.0001) for both measures. There was no main effect of genotype (p = 0.88) or interaction effect of time and genotype (p = 0.79) for food intake, and no main effect of genotype (p = 0.89) or interaction effect of time and genotype (p = 0.24) for energy expenditure. There was a main effect of sex for food intake (p = 0.0005) and for energy expenditure (p < 0.0001). Multiple comparisons were made using Šidák’s multiple comparison *post hoc* test and Tukey’s HSD test.

In the absence of this critical information, one way to explore the functional approach is by using the *BMAL1* conditional knockout to target cells expressing a specific endocrine receptor to determine the extent to which the molecular clock is necessary for system function. Leptin is an optimal endocrine candidate, not only due to its role in body weight and glucose homeostasis (for review see 24), but also due to its relationship with circadian rhythms. Evidence suggests that leptin may act as an intrinsic circadian cue, such that exogenous administration can partially rescue impaired peripheral clocks (25). Moreover, chronic circadian disruption induced by repeated shifts in the light:dark cycle is sufficient to induce cellular leptin resistance in the brain of wild-type mice (26). To explore this question, we generated a mouse model in which *BMAL1* is deleted from all leptin receptor-expressing cells (*BMAL1^+^* ^/+^;LepR^cre/?^). Due to the global nature of leptin receptor expression, this approach targets multiple cell types across the brain, adipose, pancreas, skeletal muscle, and liver with the common function of receiving the leptin hormone. We compared 13 to 23-wk old male and female *BMAL1^fl^* ^/fl^;LepR^cre/?^ mice to age- and sex-matched littermates (*BMAL1^+^* ^/+^;LepR^cre/?^) and assessed body weight, food intake, energy utilization, plasma leptin levels, and behavioral leptin sensitivity. Behavioral leptin sensitivity was determined by measuring a fasted animal’s food intake following an intraperitoneal injection of leptin or saline. Animals with high leptin sensitivity were characterized as consuming less food following a leptin injection compared to a saline injection. In comparison to controls, *BMAL1^fl^* ^/fl^;LepR^cre/?^ mice were significantly more leptin sensitive during the light phase, and significantly less leptin sensitive during the dark phase (**Figure 1**). Interestingly, this alteration in leptin sensitivity occurred despite no significant differences in diurnal leptin levels, body weight, food intake, or energy expenditure (**Figure 1**). The extent to which the molecular clock of leptin receptor-expressing cells affects glucose regulation remains unknown, but these studies are on-going.

We hypothesize that clock genes interact with elements of the leptin receptor signaling cascade to produce time-dependent alterations in leptin-induced feeding suppression. Notably, a significant increase in leptin sensitivity was observed in *BMAL1^fl^* ^/fl^;LepR^cre/?^ mice during the light phase, when *BMAL1* expression is typically high in the hypothalamus, adipose, and liver (27). Thus, in a wild-type mouse, when *BMAL1* is high, *BMAL1* may act to inhibit leptin sensitivity during the light phase. Contrastingly, the relative decrease in leptin sensitivity observed during the dark phase in *BMAL1^fl^* ^/fl^;LepR^cre/?^ mice may be due to a lack of rhythmic increase in a core clock repressor, such as *Per*, *Cry*, or *REV-ERB*.

In contrast to studies which have taken an anatomical approach, the phenotype of the present model largely indicates a selective, niche role of the molecular clock in the regulation of body weight. At least within leptin receptor-expressing cells, the molecular clock does not appear to be necessary for the regulation of day-to-day body weight, daily *ad libitum* feeding patterns, or even diurnal leptin levels – instead, the clock in this population appears to integrate timing information and fat storage information (*i.e.*, leptin) to modulate an animal’s feeding response when hungry (*i.e.*, under fasting conditions). Indeed, we find that fasted, wild-type mice exhibit substantial leptin-induced feeding suppression during their active phase in the dark. However, this same effect is not observed in mice lacking a functional clock in leptin receptor-expressing cells. We speculate that this clock-associated modulation may aid in the long-term control of body weight and optimize an animal’s feeding behavior. Hungry animals with ample fat storage may be at risk of eating in excess of their homeostatic need when presented with the opportunity to consume an abundance of calories. The clock may aid in the anticipation of such caloric abundance and increase leptin sensitivity during this time to decrease an animal’s feeding behavior. However, that same level of leptin sensitivity may be detrimental during the light phase when some minimum number of calories must be consumed to prevent the animal from entering a torpor-like state. The circadian clock of leptin receptor-expressing cells may thus aid in the anticipation of food resources and integrate that information with the animal’s fat reserves to help mount an appropriate feeding response.

This interpretation presents several new implications. First, these data suggest that clinical circadian disruption may lead to weight gain not necessarily because individuals consume more calories overall but because they specifically eat more calories following a prolonged fast. To our knowledge, this has not been tested, but the observed correlation between circadian disruption and binge-eating disorder may be relevant (28). Because our phenotype is specific to the fasted state, this also implies that circadian disruption combined with free feeding, *ad libitum* conditions would not incur weight gain. In rodents, there is some support of this notion. Circadian disruption caused by repeated shifts of the light dark cycle often has very little effect on body weight regulation when laboratory animals are allowed free access to food. However, the same paradigm combined with palatable food or food restriction leads to weight gain (for review see 29).

In addition to this study highlighting the importance of using a functional approach to gain new insights into the interactions between circadian biology and metabolic disease, this study also underscores the need for more studies to focus on leptin sensitivity – particularly at different times of day. Leptin sensitivity may prove to indicate something more than just day-to-day body weight regulation; it may prove to indicate an individual’s ability to optimize food intake when hungry. Unfortunately, there are no specified methods for evaluating leptin sensitivity in a clinical setting (30). Behavioral leptin sensitivity is even limited in basic science research and is often bypassed in favor of molecular indicators of leptin binding to its receptor (*e.g.*, pSTAT expression).

We believe the present *BMAL1^fl^* ^/fl^;LepR^cre/?^ model demonstrates the relative resilience of the peripheral tissues (*e.g*., the adipose tissue) to withstand perturbations to circadian disruption. By knocking out *BMAL1* in all leptin receptor-expressing cells, it is likely that the present model dissolves the clock in most, but perhaps not all, adipocytes (31). However, the fact that the present model fails to recapitulate the phenotype of a conditional adipose tissue knockout (18) suggests that even a minute number of adipose cells expressing *BMAL1* can compensate for clock disruption. Notably, this same resiliency cannot be said for the brain. Indeed, whether the clock is disrupted in all leptin receptor-expressing cells (as in the present model) or just in the hypothalamus (32), the resulting animal experiences a significant change in leptin sensitivity. Our observations in the hypothalamus suggest that a very small number neurons within the SCN could express the leptin receptor (33, 34). This is based on anatomical observation, not genetic expression analysis. Therefore, it remains possible that the observed effects in the *BMAL1^fl^* ^/fl^;LepR^cre/?^ mouse are specifically due to manipulation of *BMAL1* in the SCN.

Finally, while the present model did not result in any significant changes in body weight, it remains a possibility that clock disruption within leptin receptor-expressing cells will affect glucose regulation. Indeed, conditional deletion of *BMAL1* in the liver or pancreas affects glucose regulation without altering body weight (19, 21). Leptin receptor expression is found in all major liver cell types, including most if not all hepatocytes, targeted by Lamia in the conditional knockout model (35–38). Similarly, leptin receptor expression in the pancreas is well documented (19, 39). While the liver and pancreatic conditional knockouts lead to opposing effects on glucose tolerance, most of the tissue specific *BMAL1* knockout models are associated with an impairment in glucose tolerance. It remains to be determined if disrupting the clock of leptin receptor-expressing cells will recapitulate the sum of the peripheral tissue knockout models or if it will demonstrate that targeting a system as a whole is not the same as the sum of its parts.

Clinical circadian disruption is unlikely to be the result of disruption to a single tissue. Further use of a functional approach, such as the *BMAL1^fl^* ^/fl^;LepR^cre/?^ mouse, has the potential to better mirror the circadian disruption experienced by individuals. Moreover, by applying this functional approach to other systems, future research has the potential to uncover new information and mechanistic perspectives which will increase our understanding how circadian disruption impacts metabolic disease.

## 4 Conclusions

Sleep and circadian disruption are associated with detrimental effects on metabolic health. While *Per*::Luc models and the discovery of FAA have greatly advanced the circadian field, they have also inadvertently biased the field to think in terms of singular tissues and how peripheral clocks become misaligned on an individual basis. We argue that investigators can gain a better mechanistic understanding of the relationship between sleep/circadian disruption and metabolic disease by targeting groups of cells with a functional relationship. Utilizing this approach may better represent the circadian disruption experienced by individuals and may help to recapitulate the breadth of circadian disruption and its consequential effects on metabolic disease.

## Data availability statement

The original contributions presented in the study are included in the article/supplementary material. Further inquiries can be directed to the corresponding author.

## Ethics statement

The animal study was reviewed and approved by Institutional Animal Care and Use Committee of Marquette University (Milwaukee, WI).

## Author contributions

GM collected, graphed, analyzed, and interpreted all data. DA conceived/designed the experiment. GM and DA wrote and edited the manuscript. All authors contributed to the article and approved the submitted version.
